# Plants are Capable of Synthesizing Animal Steroid Hormones

**DOI:** 10.3390/molecules24142585

**Published:** 2019-07-16

**Authors:** Danuše Tarkowská

**Affiliations:** Laboratory of Growth Regulators, Centre of the Region Haná for Biotechnological and Agricultural Research, Institute of Experimental Botany, Czech Academy of Sciences, and Faculty of Science, Palacký University, CZ-783 71 Olomouc, Czech Republic; tarkowska@ueb.cas.cz; Tel.: +420-585-631-478

**Keywords:** natural sterols, plants, animals, steroid hormones, estrogens, progesterone, testosterone, boldenone, boldione, androstenedione

## Abstract

As a result of the findings of scientists working on the biosynthesis and metabolism of steroids in the plant and animal kingdoms over the past five decades, it has become apparent that those compounds that naturally occur in animals can also be found as natural constituents of plants and vice versa, i.e., they have essentially the same fate in the majority of living organisms. This review summarizes the current state of knowledge on the occurrence of animal steroid hormones in the plant kingdom, particularly focusing on progesterone, testosterone, androstadienedione (boldione), androstenedione, and estrogens.

## 1. Introduction

**Figure 1:**
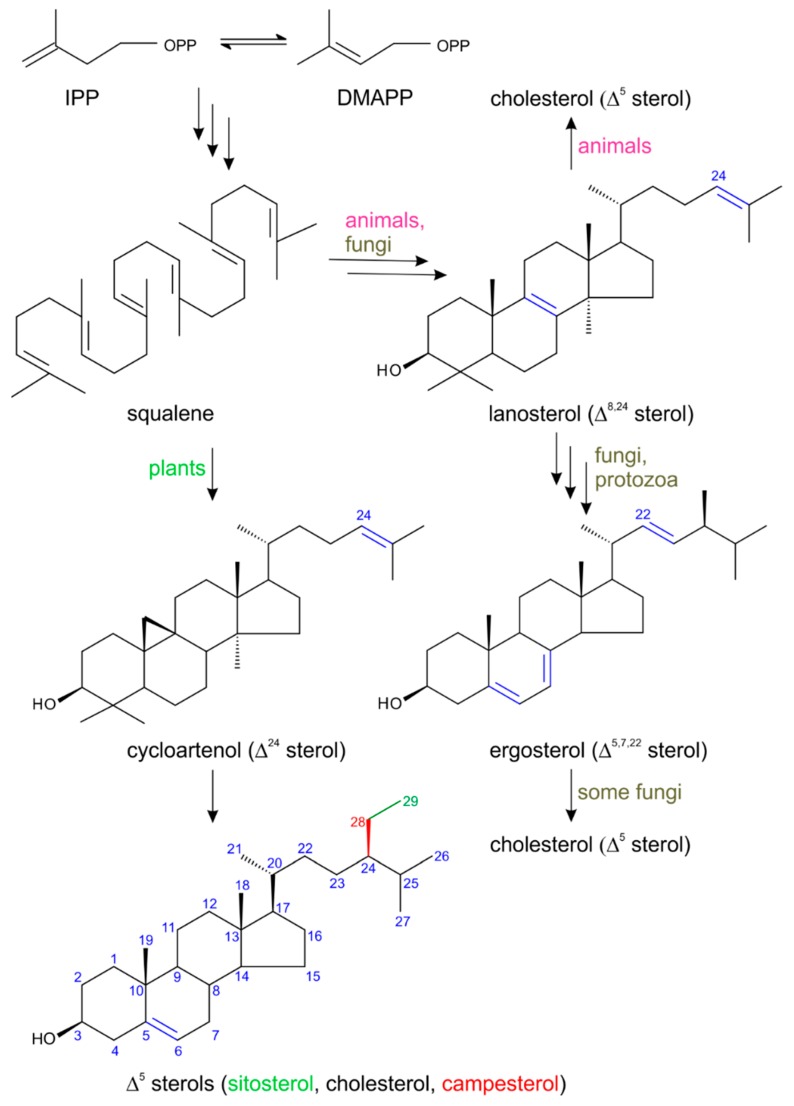
A simplified biosynthetic scheme for selected natural sterols [5,6,7,8].

The plant and animal kingdoms are not two completely separate worlds coexisting on this planet, but, on the contrary, they are two worlds whose evolution has taken place simultaneously, hand in hand with each other. It is therefore more than obvious that some substances being synthesized in nature for a particular purpose can occur in both plant and animal organisms. Certain compounds that regulate growth and development in plants may also control cellular growth and differentiation processes in animals, and vice versa. An example of such compounds may be sterols, i.e., steroid alcohols. These tetracyclic substances belong to isoprenoids, a large group of naturally occurring compounds formed in a cell by combining six units derived from the five-carbon molecule dimethylallyl diphosphate (DMAPP) and its isomer isopentenyl diphosphate (IPP) [1]. It is worth mentioning that sterols can be found in all eukaryotes (plants as well as animals), where they play many irreplaceable roles, including maintaining membrane semi-permeability, regulating their fluidity, serving as biosynthetic precursors for steroid hormones, and acting as important signaling molecules [1,2,3,4]. A typical representative of a natural sterol produced by both plant and animal cells is cholesterol (Figure 1).

This C_27_ sterol serves as a precursor of steroid plant signaling molecules (brassinosteroids, phytoecdysteroids) [1] as well as androgen- and estrogen-type sex hormones in animals [9]. When looking for the plant/animal origin of the latter hormone group, it seems that estrogens were first discovered in plants (in 1926) when Austrian medical doctor Otfried Otto Fellner, known today as a pioneer in gonadal endocrinology, was able to demonstrate estrogenic activity in oatmeal and rice [10]. At the same time, Dohrn, together with Faure, Poll, and Blotevogel, at the Anatomical Institute in Hamburg showed estrogenic activity also in other plant species including sugar beet and potato, as well as in yeast [11]. Steroid hormones with estrogenic activity were detected in the animal kingdom (in humans) about three years later by Butenandt and Doisy [12,13]. One may ask the question here if the chicken or the egg comes first, i.e., if chemical messengers, hormones, originate from plants or animals, regardless of the order in which they were discovered. Interestingly, some findings on the evolution of the neuroendocrine system in animals suggest that the hormones regulating morphogenesis and reproduction in invertebrate animals have their phylogenetic origin in more primitive multicellular organisms [14].

A recent topic of often heated discussion is the presence of the steroid hormones progesterone, testosterone and their derivatives in human diets of plant as well as animal origin. The reason for this is that a majority of these substances (except progesterone) are listed on the annually updated Prohibited list of The World Anti-Doping Agency (WADA) in the category of anabolic agents (S1) [15]. Therefore, their levels are monitored in the biological fluids of athletes and in the preparations they consume. Besides their presence in the WADA list, all the above mentioned steroids are also prohibited substances that cannot be contained in food products in European countries (according to Regulation (EC) No. 178/2002 of the European Parliament laying down the general principles and requirements of food legislation, and procedures in matters of food safety). However, the legislation does not specify for each prohibited substance at what concentration it is possibly harmful to human health, although since the sixteenth century we know, thanks to Paracelsus (1493 to 1541), that “the dose makes the poison” (Sola dosis facit venenum in Latin), i.e., a substance can cause the harmful effect associated with its toxic properties only if it reaches a biological system in a sufficiently high concentration. Therefore, regardless of their endogenous origin, once they are detected in any food product (vegetable, fruit, herb, meat) in any concentration, the seller is sanctioned for breaking the existing legislation and the food/nutritional supplement is withdrawn from sale.

The aim of this article is to point out that, in addition to well-known animal resources, steroids are also natural and integral components of plants, where they are synthesized de novo as chemical messengers for cell–cell communication, required for the regulation of physiological processes related to growth, development, and reproduction [16,17,18,19]. For this reason, it is therefore evident that steroids can be detected in plant-derived animal feed as well as in human phytosterol-rich food, and consequently in products of their secretion. This is very important information, especially for health authorities.

## 2. Biosynthesis of Plant Sterols with Respect to Steroid Hormone Formation

As mentioned in the Introduction, plant sterols (phytosterols) are a very important family of natural substances that have many biological functions in plants. It has also been known since the 1950s that they are very beneficial for humans as dietary phytosterols are able to lower levels of serum cholesterol via the inhibition of its absorption and the compensatory stimulation of its synthesis, when consumed at intake levels over 1 g per day [20,21,22]. Phytosterols may also act as precursors for the de novo biosynthesis of steroid hormones. In general, sterols are tetracyclic C_30_ terpenoid (isoprenoid) substances belonging to a group of triterpenoids [23], formed by the condensations of basic five-carbon (C_5_) building units of isoprene in the form of isopentenyl diphosphate (IPP) and dimethylallyl diphosphate (DMAPP) [1]. In higher plants, two pathways coexist to produce terpenoids—the mevalonate pathway (MVA) and the 1-deoxy-d-xylulose 5-phosphate pathway (DOXP), both named according to the first intermediate formed [1]. DOXP is also called the 2-C-methyl-d-erythritol 4-phosphate pathway (MEP) after the DOXP reduction/isomerization product in this pathway. To our current knowledge, MVA operates in the cytosol of plant cells, while the MEP pathway takes place in plastids in most eukaryotic photosynthetic organisms, but not in animals [24]. In the case of phytosterols, it has been shown that their skeleton is derived from IPP made up exclusively from acetate units via MVA in the cytosol [8,25]. The plant sterol pathway consists of a sequence of more than 30 enzyme-catalyzed steps, all of which are located in plant membranes [26,27]. The key essential intermediate in plant sterol biosynthesis is the linear C_30_ hydrocarbon squalene, which directly undergoes a cyclization to yield the C_30_ Δ^24^ sterol cycloartenol in photosynthetic plants [18]. Fungi without photosynthetic apparatus convert squalene into lanosterol and finally ergosterol [7]. Subsequent alkylation reactions of cycloartenol in photosynthetic plants lead to the synthesis of the first phytosterols (Δ^5^ sterols) such as cholesterol (C_27_), campesterol (C_28_, i.e., 24-methyl Δ^5^ sterol), and sitosterol (C_29_, i.e., 24-ethyl Δ^5^ sterol) [22] (see Figure 1). These phytosterols are starting points for the biosynthesis of the plant steroid signaling molecules phytoecdysteroids and the plant steroid hormones brassinosteroids [1], as well as progesterone, testosterone and its derivatives.

## 3. Progesterone

**Figure 2:**
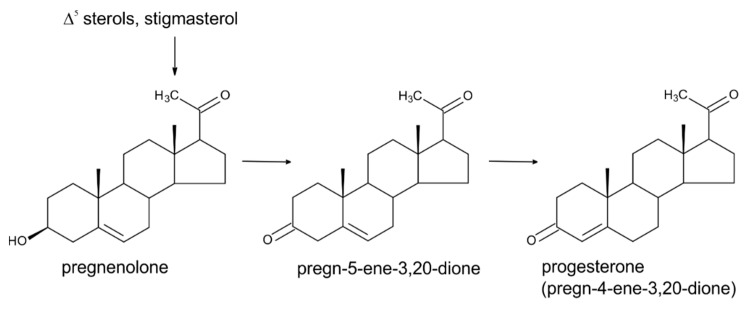
The scheme of progesterone biosynthesis in higher plants.

Progesterone (PRG; Figure 2) has been described for decades predominantly, as a mammalian gonadal hormone. Its presence in plants was reported for the first time in 1964 [28], but this finding has been questioned for many years, with claims that the methods by which it was detected in plants were non-specific and cannot be trusted [29].

At that time, thin-layer chromatography, gas chromatography, or immunoassays, were commonly used methods for the detection of natural substances in biological matrices with none or an insufficient sample purification of the crude extract [28,30,31]. However, later, with the gradual introduction of modern analytical instrumental methods (mass spectrometry), it was shown conclusively that PRG is definitely naturally present in plants [29,32,33], where it serves as a precursor in the biosynthesis of androstanes and estranes [33,34]. Numerous studies with ^3^H and ^14^C-labelled precursors showed that sitosterol, a predominant sterol in higher plants, and the less abundant sterol cholesterol can serve as precursors of PRG in plants [35,36,37,38] (see Figure 2). There are also some studies that suggest that the precursor of PRG could be campesterol and stigmasterol (C_29_, i.e., 24-ethyl Δ^5,22^ sterol), as has been well reviewed by Janeczko [19]. There is a relatively poor understanding of the biological importance/physiological functions of PRG in plants. The majority of studies have been conducted by exogenously applying PRG to various plant systems, *i.e.*, either seedlings or plant cell cultures of various plant species. Experiments of this design suggest that this substance has a certain regulatory activity in plant growth and development, influencing both vegetative and reproductive development. For instance, shoot and root growth of the common model plant Arabidopsis thaliana [29] and of sunflower [39] were demonstrated to be influenced by PRG in a dose-dependent manner. The acceleration of flowering was observed in A. thaliana and wheat exposed to micromolar concentrations of PRG [40,41]. The involvement of PRG in reproduction processes in plants has been further indicated by the PRG stimulation of tube growth of mature tobacco pollen [42], and by increasing levels of endogenous PRG during the germination of kiwifruit pollen [43]. It is worth mentioning that the authors of that study observed detectable levels of endogenous PRG and 17β-estradiol (member of the estrogen-type sex hormone family) in ungerminated kiwifruit pollen, but could not detect endogenous testosterone, while during germination (tube organization phase and subsequent elongation), the levels of PRG and 17β-estradiol dramatically increased and testosterone levels comparable with those of PRG were detected. The studies mentioned above are examples of a number of studies documenting the natural occurrence and function of these steroid substances in plants. It is, however, important to note that from the perspective of plant physiology, these compounds are not considered plant hormones by the plant science community. According to the accepted definition, for a substance to be included in the list of plant hormones, it must meet three fundamental criteria: 1. the protein–receptor for its perception must be known, 2. the compound should be omnipresent in the plant kingdom, and 3. the compound should influence physiological processes at a low concentration [44]. Some recent molecular studies dealing with steroid perception in plant cells revealed the presence of a plant membrane-localized steroid binding protein in Arabidopsis that can bind to multiple steroid molecules with different affinities, but the highest affinity was to PRG [45]. It was further shown that this protein negatively regulates cell elongation (transgenic plants overexpressing this gene have a short hypocotyl) and stimulates root gravitropism [46]. The gene for a similar binding protein from rice (*Oryza sativa* L.) was later cloned and its abundant expression described [29]. Using radioligand binding analysis, specific binding sites for PRG have been located within the cytoplasm and cell membrane of wheat [47]. A relatively widespread occurrence of PRG, together with estrogens and androgens, was demonstrated by Simon and Grinwich [31], who screened 128 plant species from over 50 families by radioimmunoassay. They found that PRG was present in about 80% of investigated species, testosterone and its derivatives in about 70% of species, and estrogens (estrone and 17β-estradiol) in about 50% of species. Interestingly, androgens (testosterone and dihydrotestosterone) were detected in the seeds of all species tested. The mass spectrometry-based reports of Iino et al. [29] and Simerský et al. [33] provided evidence that PRG, testosterone, and its derivatives are present in plant tissues in very low concentrations (pg·g*^−^*^1^ fresh weight, FW; Table 1). Thus, it may be, therefore, that all three fundamental criteria for PRG to be considered as plant hormone are, to some extent, fulfilled. Nevertheless, additional studies, including the application of PRG biosynthesis inhibitors and mutants with impaired PRG biosynthesis, are needed to understand more deeply the role of PRG and its mechanism of action in planta.

## 4. Testosterone

**Figure 3:**
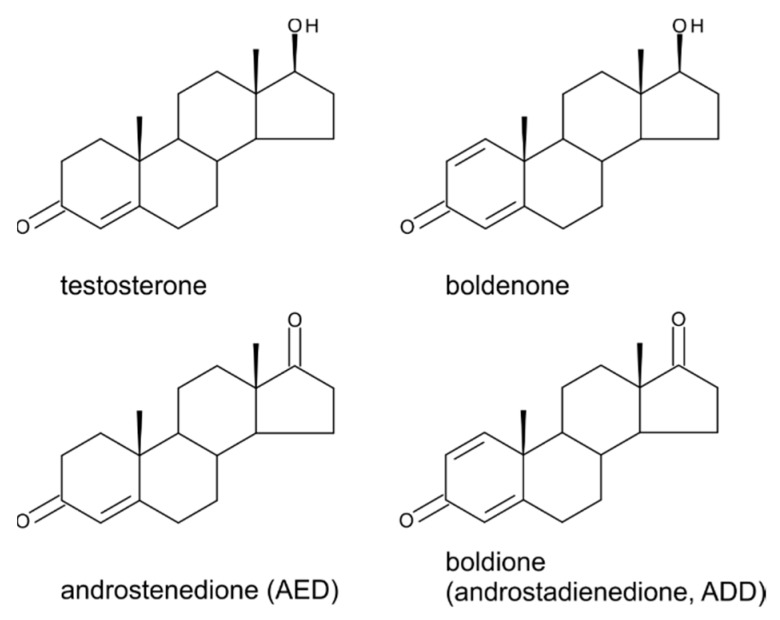
The structures of testosterone and structurally related androstanes.

If there is some reluctance to admit that steroid hormones occur in higher plants, then it is especially true for testosterone (4-androsten-17β-ol-3-one; TS; Figure 3) and its derivatives. Usually it is because people associate the effects of steroid hormones with the endocrinology of animals.

TS together with epitestosterone (4-androsten-l7α-ol-3-one) and androstenedione (4-androsten-3,17-dione) was isolated for the first time from plant sources by Šaden-Krehula et al. in 1971 [49]. The authors used pollen from Scotch pine *Pinus silvestris* and later showed the presence of all these substances as well as PRG in the pollen of *Pinus nigra* [48]. It was confirmed that *Pinus* species are a rich source of testosterone since it was further detected in *P. tabulaeformis* and *P. bungeana* and in the reproductive organs of other plant species, including ginkgo (*Ginkgo biloba*) and lily (*Lilium davidii*) [57]. Although our knowledge of the distribution of TS in plants is still fragmentary, and scientific studies dealing with its isolation and/or determination from plant sources are published very rarely, some traceable information in the literature indicates that TS and dihydrotestosterone occur in twenty plant species, including maize, barley, and rhubarb [31]. Furthermore, Hartman et al. described the natural occurrence of steroid hormones in food, demonstrating the presence of TS in potatoes, soybeans, haricot beans, and wheat, where its levels ranged between 0.02 and 0.2 μg⋅kg^−1^ [50] (Table 1). The authors reported that this androgen also occurs in native oils used in human nutrition, such as olive oil, corn oil, and oil made from safflower (*Carthamus tinctorius*) seeds. Interestingly, safflower oil has been shown to lower lipids and lipoproteins in human serum due to its high content of linoleic acid (73% to 77%) [58], which belongs to the highly important omega-6 unsaturated fatty acids group and is an essential fatty acid in the human diet. Linoleic acid is also known as vitamin F. Safflower is also valuable for beekeepers as a melliferous plant with a high nectar content in late summer as well as being a promising source for biodiesel production, i.e., one of the renewable energy sources [59]. The phytosterol content of safflower is relatively high, ranging between 2000 and 4500 μg⋅g^−1^ in seeds, in which β-sitosterol accounts for the largest percentage (50% to 70%) of the total phytosterol content [60].

**Figure 4:**
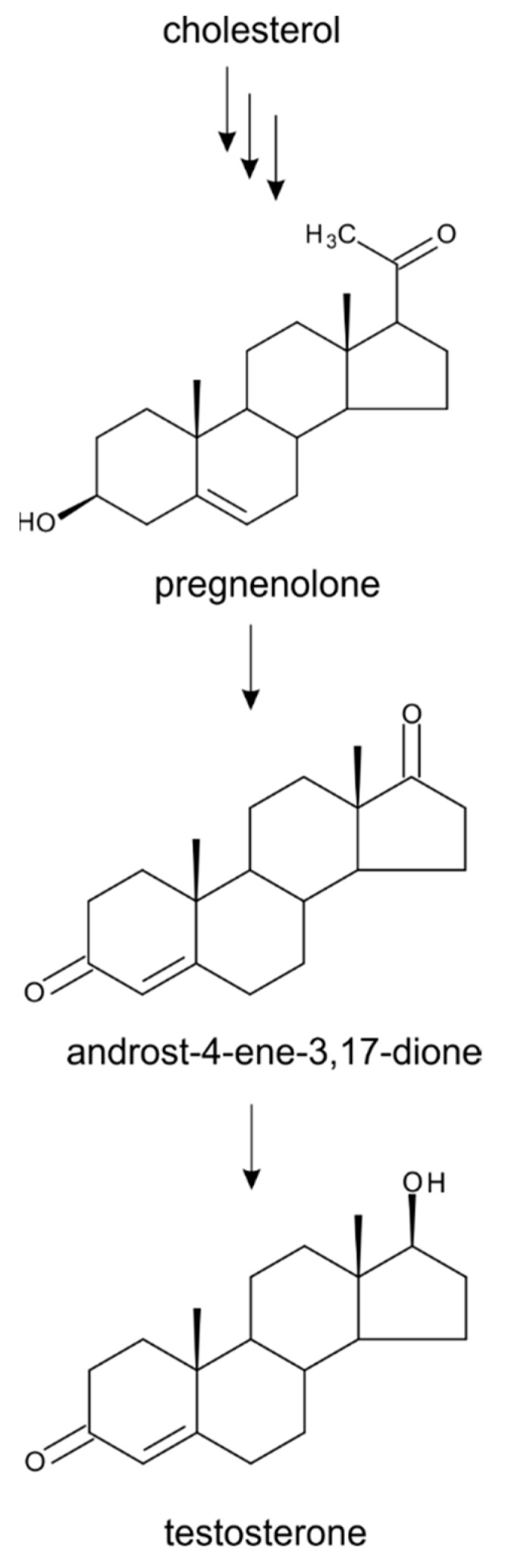
**A** simplified biosynthetic pathway of testosterone in plants.

From a biochemical standpoint, the biosynthesis and biological function of TS in plants seem to be similar to those in animals [16,61,62]. This C_19_ steroid is formed via the MVA pathway in the cytosol of plant cells from cholesterol by the sequential action of multiple enzymatic reactions. These reactions include the side chain cleavage of cholesterol to the C_21_ steroid pregnenolone, followed by a transformation into androstenedione and finally into TS (Figure 4). The latter enzymatic step was confirmed by feeding experiments with ^14^C-androstenedione, which was converted to TS in pea and cucumber seedlings and in cultured cells of *Nicotiana tabacum* [63,64,65]. In contrast to the animal kingdom, where TS and other androgen substances act only as sex hormones, it has been shown in plants that they affect not only their reproductive development (especially flowering and floral sex determination), but also vegetative development [17,18].

## 5. Boldenone, Boldione, and Other Testosterone Derivatives

Boldenone (Bol) is the trivial name for 1-dehydrotestosterone or androsta-1,4-diene-17β-ol-3-one (Figure 3). Androsta-1,4-diene-3,17-dione (ADD) and androst-4-ene-3,17-dione (AED) are closely related metabolites—Figure 3. ADD (androstadienedione) is recognized as a Bol precursor in various animal species including humans and is trivially named as boldione, whereas AED is considered as a TS precursor [66] and can be found in the literature often as androstenedione rather than its full name or AED. Both Bol and boldione are prohibited anabolic steroids in European Union Member States. As in the case of estrogens, PRG and TS, there are still discussions about whether these substances are detected in surveillance laboratories as a result of illegal direct administration or whether they are of endogenous origin. These discussions concern both animal and human samples as well as plant-based food supplements, in which their positive detection attracts special attention. In the vast majority of cases, a positive sample of a nutritional supplement is considered to be harmful to health because it is assumed to be deliberately enriched with a forbidden anabolic steroid. There are, however, scientific studies that admit that both weak androgenic substances of interest (Bol and boldione) can be formed endogenously from phytosterols and may thus occur naturally in plants [67]. If we look for plant sources where either ADD and/or AED was detected, we find that AED was determined in pine pollen of *P. sylvestris* (0.59 µg·g^−1^) and *Pinus nigra* (0.08 µg·g^−1^), using various analytical methods, as far back as in 1971, 1979, and 1983 [48,49,52]. Later, in 1998 relatively significant amounts of AED were quantified in the important foodstuffs – wheat (0.48 ng·g^−1^) and potato (0.05 ng·g^−1^) [50]. Quantitation data are summarized in Table 1. Trace quantities were further observed in soybeans, haricot beans, mushrooms, olive oil, and safflower oil, as well as in wine and beer. With increasing sensitivity, i.e., the ability to reach lower limits of detection (LOD)/limits of quantitation (LOQ), AED was determined in other plant species such as tobacco (*Nicotiana tabacum*; 2.20 ng·g^−1^ FW) and the native European herb elecampane (*Inula helenium*; 3.20 ng·g^−1^ FW) [33]. AED and ADD together with PRG and TS were unequivocally detected in the annual creeping herbaceous plant *Tribulus terrestris* by very sensitive and precise analysis based on ultra-high performance liquid chromatography–tandem mass spectrometry (Tarkowská, unpublished). *T. terrestris* is widespread globally and it is used in folk medicine, mainly for the treatment of cardiovascular and eye diseases and for high blood pressure. In Europe and the USA, food supplements containing *T. terrestris* extracts are on sale as regenerative/adaptogenic agents similar to ashwagandha (indian ginseng; *Whitania somnifera*) or ginseng (*Panax ginseng*) [68]. The highest concentrations of the four investigated steroids in *T. terrestris* were found for PRG, with levels in plant tissues ranging from 0.01 to 0.015 µg·g^−1^ of dry weight. TS and AED were present at approximately the same level and reached about 0.25% of PRG levels. AED levels were an order of magnitude lower than TS levels. The lowest levels were found for ADD, which generally was present at one tenth to one hundredth of the concentration found for AED (Table 1). Recently, phytochemical analysis of the crude extracts isolated from *Pinus halepensis* needles revealed the presence of ADD [51]. Thus, taking into account all the above-mentioned facts, it is clear that these androgen substances, which were assumed to be animal-derived, may also have a plant origin.

The effect of androgens on physiological processes has been best investigated in humans and other animals. To investigate their functions in plants, similar experimental designs have been applied to several plant species. Plant growth was affected in Arabidopsis and winter wheat (*Triticum aestivum*), where the TS precursor AED stimulated the proliferation of callus tissue and promoted the germination and growth of immature embryos, respectively [69,70]. However, it is unfortunate that these data are difficult for the general scientific community to access. There is also evidence that AED can influence developmental processes associated with plant reproduction, such as flowering. In some plant species (Arabidopsis, wheat), AED treatment caused a significant increase in the percentage of plants reaching the reproductive stage [40,41]. Experiments examining the metabolism of androgens in higher plants (pea and cucumber) showed that ^14^C-labeled AED added to leaves was transformed to TS [63,64]. These results are in agreement with those found in animal cells. Therefore, it seems that plant cells might have a similar enzymatic apparatus for the biosynthesis and metabolism of androgen substances as that of animal cells.

## 6. Estrogens

**Figure 5:**
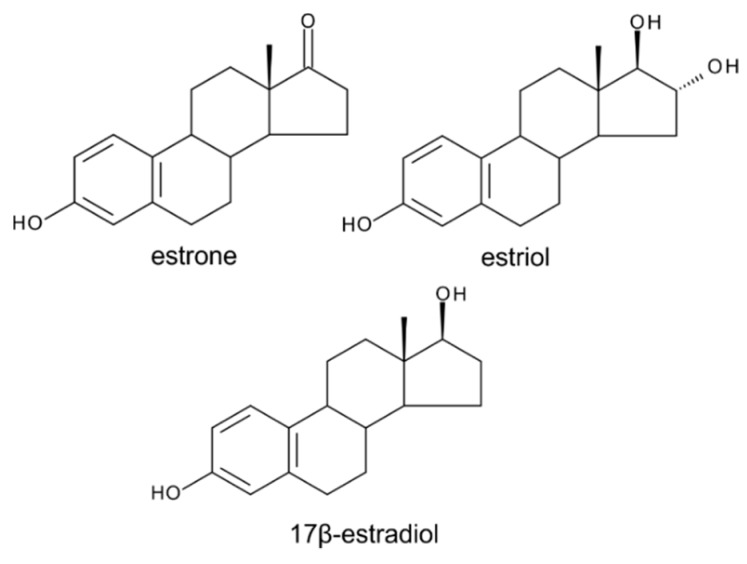
The structures of selected estrogens.

Estrogens (Figure 5) belong to steroid-type substances commonly referred to as animal sex hormones since they are produced by ovaries in adult females of higher vertebrates, in which they are responsible for the development and regulation of the female reproductive system.

However, the observation of estrogenic activity was first made in plant extracts (late 1920s) even before structure elucidation of the endogenous animal estrogens [11,71]. Estrogenically active substances were subsequently isolated in crystalline form from palm kernels [72] and willow catkins [73]. Various authors later confirmed the presence of steroidal estrogens in many plant species including date palm, bean, pomegranate, and the species of the *Prunus* genus [53,56,74,75,76,77,78]. Although numerous authors have described the presence of steroidal estrogens as well as their biosynthetic pathway, some authors have, historically, disputed their presence in plants [79,80]. Nevertheless, in 2001, estrogen receptor-like proteins were isolated from various plant organs and shown to be localized in the nucleus [81]. Furthermore, there are certain indications that steroidal estrogens affect growth and reproduction in plants [14,18]. For instance, they have been shown to stimulate embryo and seedling growth in pea [82,83], sunflower [39], and tomato [84]. Analogously to their reproductive function in animal kingdom, steroidal estrogens were reported to also influence plant reproductive development such as flowering and sex determination of flowers. Already in 1937, Chouard demonstrated the flower promoting effect of estrogens by watering the plants of *Callistephus sinensis* with 17β-estradiol (Figure 5) solution [85]. Later, the induction of flowering by estrogens was achieved also in very diverse plants growing under non-inductive conditions. In common chicory *Cichorium intybus*, the inductive cold period could be substituted by 17β-estradiol or estrone (Figure 5) treatment [86]. Similarly, the flowering of scarlet sage *Salvia splendens*, which is naturally initiated under long day conditions, was induced by 17β-estradiol and by unidentified estrogen-like substance isolated from flowering *Salvia* plants under short day conditions [87]. According to some scientific reports, the application of estrogens can considerably affect sex determination of flowers of plants that have both male and female flowers, i.e., dioecious plants. The ratio of female to male flowers was significantly affected by the application of estrogens (estrone, estriol, 17β-estradiol) when the percentage of female flowers increased by 66% in *Ecballium elaterium* L. while the total number of flowers was enhanced by 18% to 35% [88]. 17β-estradiol can be used to modulate the development of flowers to male or female, which has been shown in cucumber *Cucumis sativus*. The treatment of cucumber plants with 0.1 mg of 17β-estradiol induced a formation of an increased number of female flowers by about 20% [89].

Considering the above-mentioned findings, it is not surprising that the levels of endogenous steroidal estrogens are highest in reproductive parts of the plant, such as flowers, pollen grains, fruits, and seeds, whereas vegetative organs (stem, leaves, roots) are poorer sources of these substances [14]. As mentioned above, estrogens were detected in 50% of 128 species screened [31] so they can be considered as widespread naturally occurring substances—see Table 1.

Regarding their formation in cells of higher plants, it is assumed that the precursor of steroidal estrogens is cholesterol formed via the MVA pathway, i.e., the biosynthetic pathway of estrogens in plants is similar to that known in animals [16]. This hypothesis has been supported by experiments with radiolabeled precursors, from which it was observed that 17β-estradiol was formed from [2-^14^C] mevalonic acid in bean seedlings [56]. The same authors demonstrated the conversion of estrone to 17β-estradiol in the same plant species [90]. They proposed that enzymes capable of this conversion were located in leaf tissue.

## 7. Conclusions

The data discussed in this review provide evidence of the ability of plants to convert sterols into steroid hormones. This applies both to the plant steroid hormones brassinosteroids, as well as to other steroid substances, such as phytoecdysteroids, estrogens, progesterone, and androgenic substances of the testosterone type, including testosterone precursors and derivatives. Although it was reported here that all classes of animal steroids have been found in plants, this does not necessarily mean they are hormonally active.

## Figures and Tables

**Table 1:** The results of quantitative analysis of selected steroids in plant material.

Steroid	CAS Number	Mol. Formula	Mol. Weight (g·mol^−1^)	Amount	Origin	Ref.
progesterone	57-83-0	C_21_H_30_O_2_	314.46	0.08 µg·g^−1^	*Pinus nigra*	[48]
				3–1600 ng·g^−1^	31 plant species	[31]
				6–1540 ng·kg^−1^	8 plant species	[29]
				1.19–15.5 µg·g^−1^	*Pinus taeda*	[32]
				0.66 ng·g^−1^	*Inula helenium*	[33]
				17.4 ng·g^−1^	*Nicotiana tabacum*	[33]
				18.5 ng·g^−1^	*Digitalis purpurea*	[33]
				0.02–15.39 ng·g^−1^	*Tribulus terrestris*	Tarkowská, unpublished
testosterone	58-22-0	C_19_H_28_O_2_	288.42	0.08 µg·g^−1^	*Pinus silvestris*	[49]
				0.09 µg·kg^−1^	wheat	[50]
				0.05 µg·kg^−1^	corn oil	[50]
				0.21 µg·kg^−1^	safflower oil	[50]
				0.01–0.02 ng·g^−1^	*Tribulus terrestris*	Tarkowská, unpublished
androsta-1,4-diene-3,17-dione (boldione)	897-06-3	C_19_H_24_O_2_	284.40	not quantified	*Pinus halepensis*	[51]
				0.1–2.7 pg·g^−1^	*Tribulus terrestris*	Tarkowská, unpublished
androst-4-ene-3,17-dione	63-05-8	C_19_H_26_O_2_	286.41	0.59 µg·g^−1^	*Pinus silvestris*	[49]
				0.09 µg·g^−1^	*Pinus nigra*	[48]
				0.08 µg·g^−1^	*Pinus nigra*	[52]
				0.05 ng·g^−1^	potato	[50]
				0.48 ng·g^−1^	wheat	[50]
				2.20 ng·g^−1^	*Nicotiana tabacum*	[33]
				3.20 ng·g^−1^	*Inula helenium*	[33]
				0.01–0.05 ng·g^−1^	*Tribulus terrestris*	Tarkowská, unpublished
estrone	53-16-7	C_18_H_22_O_2_	270.37	2.5–4.5 µg·kg^−1^	pomegranate	[53]
				5.13/5.25 µg·g^−1^	*Haphaene thebaica*	[54]
				0.04 µg·ml^−1^	*corn oil*	[54]
				33.75 µg·g^−1^	*Olea europea*	[55]
				28–420 ng·g^−1^	20 plant species	[31]
17β-estradiol	50-28-2	C_18_H_24_O_2_	272.38	2–10 µg·kg^−1^	*Phaseolus vulgaris*	[56]
